# Public and Private Hospital Nurses’ Perceptions of the Ethical Climate in Their Work Settings, Sari City, 2011

**DOI:** 10.17795/nmsjournal12867

**Published:** 2014-04-17

**Authors:** Ali Asghar Ghorbani, Ali Hesamzadeh, Mohammad Khademloo, Salimeh Khalili, Shamim Hesamzadeh, Valerie Berger

**Affiliations:** 1 Deputy of Education, Mazandaran University of Medical Sciences, Sari, IR Iran; 2 Community Health Nursing Group, Nasibeh Nursing and Midwifery School, Mazandaran University of Medical Sciences, Sari, IR Iran; 3 Social Medicine Group, Sari Medical School, Mazandaran University of Medical Sciences, Sari, IR Iran; 4 Nursing Office,Valiaser Hospital, Qaemshahr, IR Iran; 5 Statistics and Technology Office,Welfare Organization, Sari, IR Iran; 6 Nursing Department, Lone Star College, Houston, Texas, The USA

**Keywords:** Hospitals, Public, Hospitals, Private, Nurses

## Abstract

**Background:**

Nurses’ perceptions of ethical climate patterns have certain undeniable effects on hospitals. There is little evidence of possible differences in this element between public and private hospitals and contributing factors.

**Objectives:**

This study investigated whether the perceptions of the ethical climate in nurses’ working in public hospitals differ from that of nurses in private hospitals, and which factors may affect nurses’ perceptions.

**Materials and Methods:**

A cross-sectional study of randomly selected registered nurses (n = 235), working in four public hospitals affiliated to Mazandaran University of Medical Sciences, and three private hospitals, was conducted in Sari City, Iran. A self-administered questionnaire, containing demographic characteristics and the Hospital Ethical Climate Survey (HECS), were used to assess registered nurses’ perceptions of public and private hospitals ethical climate. An independent t-test and one-way ANOVA were used to analyze the data.

**Results:**

Across the five factors of HECS, the highest and lowest mean scores pertained to managers and physicians, respectively, in both public and private hospitals. Nurses who had a conditional employment situation and those working in pediatric intensive care units showed significantly more positive perceptions of the ethical work climate when compared to their peers (P < 0.05). Although the mean score of ethical work climate in private hospitals (3.82 ± 0.61) was higher than that in public hospitals (3.76 ± 0.54), no significant difference was found (P = 0.44).

**Conclusions:**

Hospital managers need to discover better ways to promote safety and health programs for their staff according to nurses’ area of work and their type of units. They should also encourage greater levels of participation in safety-enhancing initiatives in the hospital’s ethical climate, especially in the areas of nurses’ perceptions of their physician colleagues, and for nurses with a conditional employment situation.

## 1. Background

Ethics are defined as underlying principles that infuse laws, social customs and codified rules of professional groups (1). It is the area of philosophy that deals with the values and customs of a person or society; essentially, how one determines what is right or wrong (2-4). The idea that an organization has a distinct and identifiable ethical climate has been a topic of investigation in the business literature since the 1960s, and it has longstanding conceptual robustness and operation capability (5, 6). The construct of the ethical work climate was first introduced in 1987 (3, 7-12). Then, moral development and sociocultural theories of organization were used to investigate the different types of ethical climate within an organization (13, 14). Ethical climate is defined as the pervasive moral atmosphere of a social system, characterized by shared perceptions of right and wrong as well as common assumptions about how moral concerns should be addressed (6). Organizational climate symbolizes what an organization truly values, and this is revealed through the shared perception of employees, such as the kinds of behaviors that are encouraged, supported and rewarded within the organization. This shared perception of accumulated expectations and corresponding rewards, serves as an effective frame of reference for guiding the behaviors of employees (3).

Evidence suggests that among organizational variables, such as turnover intention, job satisfaction, organizational commitment and moral awareness, employees’ ethical behavior is influenced to a large extent by their perceptions of the organizational climate (13, 15-21). Ethics and the ethical aspects of health care delivery are important topics of concern for the health care industry, hospital administrators, and nurses. Interest in nursing ethics has been fueled by increased awareness of the complexity of ethical issues in health care, as revealed in the media, as well as those confronted by nurses and other health care professionals in the workplace (22). The pace of change in the health care environment continues to accelerate, and within the health care system the hospital is especially vulnerable to such changes (23).

In Iran, primary health care services are basically delivered by the public sector and are almost free of charge, particularly for those on low incomes. Services like specialty and super-specialty curative services are areas in which both public and private sectors play distinctive roles (24). The private sector mainly focuses on secondary and tertiary health care in urban areas (25). In 2006, there were 505 public institutes with 79772 beds and 128 private institutes with 12594 beds in the country (26). It is important to note that the private health care sector is growing rapidly in many countries. Gross heterogeneity of private providers in different settings has also been noted (27). Today’s health care environment has increased its appreciation of the perceptions that nurses have of their work environment or organizational climate, by involving nurses in decision-making involving ethical issues, and valuing their role when ethical questions and problems arise, as well as when implementing decisions related to ethical issues (2). A previous study of ethical work climate recognized that ethical behavior is a function of organizational issues and managerial practices, as much as it is a function of personal values (9). Olson in her ethical climate survey of hospital nurses found that the subscales of peers and physicians had the highest and lowest scores, respectively (22); on the other hand, Pauly et al. reported that peers and hospital subscales had the greatest and lowest ratings, respectively (28). In contrast, a study by Mobasher et al. showed that the highest and lowest scores belonged to managers and hospital subscales, respectively (29). There is lack of research on the ethical climate status and its subscales, together with its related factors in both public and private hospitals, and there has been no previous comparison made between these two settings with regard to nurses’ perceptions of the ethical climate and its factors, as well as influencing elements.

## 2. Objectives

The aim of this study was to compare the total ethical climate and its factors as perceived by nurses working in public and private hospitals, and to detect the influencing elements of their perceptions.

## 3. Materials and Methods

A cross-sectional study was conducted to determine how nurses in private and public hospitals perceived the ethical climate in their work environment in Sari City, during 2011. Krejcie and Morgan’s equation was used to determine the sample size required (30):

n = Z^2^ NP(1− P) ÷ d^2^ (N −1) + Z^2^ P(1− P),

according to the following parameters: Z = 1.96, N = 871 (total number of registered nurses in all public and private hospitals), P = 0.5 (maximum possibility) and d = 0.053, the sample size was then calculated, which was equal to 245, or approximately 250 nurses. The sample size for each hospital was calculated according to the proportion of the total number of registered nurses (RNs) in each hospital, to the total number of RNs in all hospitals. The questionnaires were distributed by the researchers with the help of hospital nursing managers and the question sheets were collected two days later. The 250 questionnaires were distributed to a randomly selected sample of 871 working registered nurses in all four public educational hospitals with a total of 718 RNs, and all three private hospitals with a total of 153 RNs in Sari City, Iran, from September to November 2011. The nurses were randomly selected from the list of working nurses in each hospital by using a table of random numbers.

Inclusion criteria for participation in the research were; holding a nursing registration and having at least one year’s experience in the given hospitals’ units. Informed consents were obtained from the participants after ensuring them of the study’s anonymity, and privacy.

Demographic characteristic data including; gender, age, years of work experience, educational level, place of employment, and employment conditions were collected in the first part of the questionnaire. The nurse’s level of perception of the ethical climate was evaluated by using the Hospital Ethical Climate Survey (HECS) questionnaire, with 26 items composed of five factors: peers (four items), patients (four items), managers (six items), hospital (six items), and physicians (six items). This questionnaire was first developed by Olson in 1995 (22, 31). The HECS instructs participants to answer the questions in a Likert-type format with all items rated from 1 to 5 (1 = ‘not true’ to 5 = ‘always true’) with higher values indicating a more favorable ethical climate. The minimum and maximum scores of the questionnaire were 26 and 130, respectively.

In Iran, the HECS was first translated into Farsi by Mobasher et al. during 2005 in Kerman University of Medical Sciences, and its validity and reliability was tested with a Cronbach's alpha of 0.92, which showed satisfactory results (29). In our research, the questionnaire’s face validity was proved to be desirable by a panel of ten nurses with master and doctoral degrees in nursing, who were working in both clinical and academic settings. In the pilot study, the internal consistency reliability using a Cronbach’s alpha, was calculated for the entire HECS and the result was an alpha of 0.88. Construct validity was supported in the factor analysis. The Kaiser-Meyer-Olkin measure was 0.91 denoting sampling adequacy (32). Bartlett’s test of sphericity was statistically significant (P < 0.0001). Factor analysis of the scale was carried out using principal component analysis using the Varimax method (2), and the acceptable level for scale items was set at above 0.41. All items demonstrated moderate to strong loading.

### 3.1. Ethical Considerations

This study was a research project that was approved by the Deputy of Research and Technology of Mazandaran University of Medical Sciences, and it complied with ethical considerations. The researchers were officially introduced to the hospital managers, and before questionnaire distribution, informed consent was obtained from all nurses who participated in the research, along with assurances that their answers would remain confidential.

### 3.2. Data Analysis

Descriptive and analytical statistical tests were performed, using SPSS (version 11.5). Significant statistical conclusions were determined at P < 0.05. For appropriate variables, measures of central tendency (eg, mean) and dispersion (eg, standard deviation) were described. The rates of some categorical variables were also reported. In order to compare the means of the questionnaires’ total scores in two groups or more, a t-test and one-way ANOVA were used.

## 4. Results

Fifteen questionnaires were excluded from the survey due to incomplete answers and the final sample consisted of 235 completed survey forms. Of the 235 nurses who participated in the research, there were 195 (83%) females and 40 (17%) males, with 168 (71.5%) working in public educational hospitals and 67 (28.5%) in private hospitals. Among the work experience categories, the majority, 88 nurses (37.4%), had 1 - 4 years of experience, and among the hospital units the majority, 111 (47.0%) nurses, had been working in general units (including orthopedic, surgical and medical), 17 (7.3%) in emergency units, 17 (7.3%) in pediatric intensive care units, and 90 (38.4%) had been working in adult intensive care units (Table 1). Nurses who worked in pediatric intensive care units perceived the ethical climate more positively than those in other wards of both private and public hospitals (F = 1.92, P = 0.02).

In the private hospital as well as in the total sample of both hospitals, nurses’ employment conditions were found to have a significant effect on their perceptions of the ethical climate (Table 2). Nurses with conditional employment states showed better perceptions of their work ethical climate than nurses with other employment conditions in the private hospitals (F = 4.48, P = 0.01) and in the total sample (F = 2.81, P = 0.032).

Other demographic variables, including; gender, age, years of experience, level of education, and area of work, were not significantly correlated with the mean score of ethical climate, as measured by the HECS. The means for each of the five factors of the HESC (peers, patients, managers, hospital and physicians), for both public and private hospitals, were calculated (Table 3). Among these factors, the physicians’ subscale had the lowest mean score in both public and private hospitals, while the managers’ subscale obtained the highest score. Private hospital nurses perceived the ethical climate of their work environments more positively than public hospital nurses, but the difference was not statistically significant (P = 0.44). The highest and the lowest mean scores of the HECS items, as well as items that were significantly different between the two groups of hospitals, are shown in Table 4. Managers and physicians factors had the highest and lowest scores, respectively. A t-test demonstrated that there were significant differences between some of the HECS items in public hospitals and private hospitals, concerning nurses’ perceived ethical climate in the related factors of patients (P = 0.045), managers (0.035) and physicians (P = 0.02).

**Table 1.** Comparison of Demographics of Nurses Working in Public and Private Hospitals

Demographics	Public Hospitals	Private Hospitals	Total	Statistical Indicators
**Gender, %**				
Female	81.4	86.6	83	χ^2^ = 0.89,P = 0.35
Male	18.6	13.4	17	
**Age, mean ± SD, y**	31.02 ± 6.59	36.57 ± 6.7	32.61 ± 7.08	t = 5.77, P = 0.001
**Nursing education, %**				χ^2^ = 5.39, P = 0.07
Associate	1.8	7.5	3.4	
Baccalaureate	97	92.5	95.7	
Master	1.2	0.0	0.9	
**Work experience, %**				χ^2^ = 12.39, P = 0.002t
1 - 4 Years	44.6	20.9	37.4	
5 - 9 Years	30.7	35.8	32.6	
10 ≤ Years	24.7	43.3	30.0	
**Employment condition, %**				χ^2^ = 43.43, P = 0.001
Official	24.0	52.2	32.1	
Contractual	24.6	4.5	18.8	
Conditional	26.3	43.3	31.2	
Man Power Plan	25.1	Not applicable	17.9	

**Table 2.** Mean Scores of Total HECS in Public and Private Hospitals According to Nurses Employment Conditions ^a^

Employment Condition	Public Hospitals	Private Hospitals	Total
**Official**	3.74 ± 0.47	3.62 ± 0.71	3.67 ± 0.60
**Contractual**	3.67 ± 0.6	3.72 ± 0.29	3.66 ± 0.58
**Conditional**	3.81 ± 0.56	4.08 ± 0.35	3.92 ± 0.5
**Man Power Plan**	3.79 ± 0.55	Not applicable	3.79 ± 0.55
**P value (ANOVA)**	(F = 0.54, P = 0.68)	^a^(F = 4.48, P = 0.01)	^a^(F = 2.81, P = 0.032)

^a^ Significant difference between the employment conditions

**Table 3.** Comparison of Mean Scores of Hospital Ethical Climate Factors Between Public and Private Hospitals Using a t-test

HECS ^a^ Factors	Mean Score ± SD		T (P value)	95% Confidence Interval of Differences
	Public Hospitals	Private Hospitals		
**Peers**	4.12 ± 0.5	4.05 ± 0.64	0.60 (0.55)	-0.11, 0.20
**Patients**	3.94 ± 0.64	4.01 ± 0.65	-0.74 (0.46)	-0.25, 0.11
**Managers**	4.23 ± 0.77	4.24 ± 0.76	-0.21 (0.83)	-0.25, 0.20
**Hospitals**	3.37 ± 0.81	3.48 ± 0.88	-0.90 (0.36)	-0.35, 0.13
**Physicians**	3.31 ± 0.76	3.46 ± 0.73	-1.33 (0.18)	-0.36, 0.07
**Total**	3.76 ± 0.54	3.82 ± 0.61	-0.77 (0.44)	-0.23, 0.10

^a^ Abbreviations: HECS, Hospital Ethical Climate Survey

**Table 4.** Highest and Lowest Mean Scores of HECS Items and Items With Significant Differences in Public and Private Hospitals

HECS Items (related factor)	Mean Score ± SD		T (P value)
	Public Hospitals	Private Hospitals	
**Manager listens to me (managers)**	4.23 ± 0.84	4.48 ± 0.77 ^a^^b^	-2.11 (0.035)
**Manager is someone I respect (managers)**	4.38 ± 0.81 ^a^	4.31 ± 1.10	0.53 (0.60)
**Physicians ask nurses’ opinions (physicians)**	2.74 ± 1.06 ^c^	3.00 ± 1.14	-1.60 (0.11)
**Nurses and physicians have respect for each other (physicians)**	2.83 ± 1.17	2.95 ± 1.12 ^c^	-0.66 (0.50)
**The patients’ wishes are respected (patients)**	4.27 ± 0.83 ^b^	4.40 ± 0.99	-2.01 (0.045)
**My manager listens to me talk about patient care issues/problems (managers)**	4.23 ± 0.84 ^b^	4.48 ± 77	-2.11 (0.035)
**Nurses and physicians trust one another (physicians) **	3.52 ± 0.95 ^b^	3.97 ± 0.95	-3.19 (0.02)

^a^ The highest mean score in each hospital

^b^ Significant difference between two hospitals (P < 0.05)

^c^ The lowest mean score in each hospital

## 5. Discussion

This study was conducted to compare the ethical climate and its factors as perceived by nurses working in public and private hospitals. The results show that demographic characteristics (such as; gender, age, years of work experience and educational level) do not influence the perceptions of nurses in regard to the ethical climate. Mobasher et al. did not find any significant relationship between the demographic attributes of nurses, including; age group, gender, years of work experience, education, and job position (29). Borhani et al. also found no relationship between several dimensions of their ethical climate scale and some demographic features of hospital nurses (33). Nevertheless, other researchers have suggested that there is a partial correlation between demographic features and levels of ethical climate perception in nurses, and these demographic features include; age and years of experience (2, 7). The predominant ethical climate is phenomenologically external to the person (34) and it has been recognized that individual characteristics alone are insufficient to explain moral and ethical behaviors (14), thus further research needs to be carried out on the relationship between nurses’ demographic characteristics, especially in different hospital units, and employment conditions, and their perceptions of their work ethical climate.

Private hospital nurses’ perception of ethical climate was more positive than that of their counterparts in public hospitals, though this difference was not significant. In a study in Turkey, the researchers reported that private hospital nurses perceived the ethical climate in their organizations more positively than nurses working in other hospitals. They concluded that private hospitals perform studies on quality and standards, patient satisfaction, and patient rights, in order to attract more customers, provide better quality units, and improve the existing units; in addition, private hospital organizations establish their objectives and policies by giving greater priority and importance to ethical behaviors (2).

Wittmer and Coursay also found no difference between the level of managers’ perception of public and private organizations regarding ethical climate, and they suggested that further empirical testing should be conducted, both to develop ethical climate measures and to confirm sector differences (9). The highest and lowest mean scores of the items rated by both public and private hospitals belonged to managers and physicians HECS factors, respectively. The results are in agreement with the findings of a previous report showing that the physicians factor obtained the second lowest mean score (28). Mobasher et al. indicated similar results in their research and they proposed that cooperation between nurses and physicians should be improved, otherwise it will have negative effects on patients’ outcome (29). It is evident that conflicts with doctors are a source of stress and the main cause of job dissatisfaction for nurses (35). An understanding of the concept of ethical climate should help nurses and administrators to effectively manage and create a more desirable ethical climate in their work settings, and to identify the interventions needed to change or to create conditions whereby ethical issues and problems can be raised, discussed, and resolved (22).

In the present study, the sample size of private hospitals was relatively small and this could limit the generalization of the results to other nurses in private hospitals. Future research should examine the perceived ethical climate among nurses with larger sample sizes and include other health care professionals, such as physicians, physiotherapists and laboratory staff. Results of this study further emphasize that public and private hospitals managers should develop strategies to improve relationships between peers, managers, hospitals, patients and physicians. Reinforcement of ethical climate norms has certain undeniable benefits for hospitals. It has been demonstrated that ethical climate perceptions have the power of facilitating both positive and negative organizational outcomes and evidence suggests that an organization’s ethical environment influences employees’ decisions to comply with safety rules and participate in safety-enhancing initiatives. Nurses hold positions of responsibility, which include nurse-patient relationships, effective interventions, and resource management. The nurse, in each of these situations, is responsible to both patients and the public to provide unique professional expertise and organizational influences on individuals regarding the ethical climate.

The results of this study demonstrated that nurses’ personal characteristics, including; gender, age, years of experience and education level, do not influence the ethical climate. In contrast, environmental features, such as the nature of the hospital (private or public), employment condition and type of unit, underpin the ethical climate. Hospitals, especially in the public sector, should pursue positive practices which are compatible with ethical principles and professional ethical behaviors within their institutions, while expectations and deficiencies should also be identified and improved.
